# Profile of Serum Heat Shock Protein-27 Level in Patients with Salivary Gland Tumor

**DOI:** 10.30476/DENTJODS.2021.89066.1384

**Published:** 2022-09

**Authors:** Negar Moghadasi, Azadeh Andisheh-Tadbir, Amin Samiee, Shima Torabi Ardekani, Bijan Khademi, Mahyar Malekzadeh, Razieh Zare

**Affiliations:** 1 Undergraduate Student, School of Dentistry, International Branch, Shiraz University of Medical Sciences, Shiraz, Iran; 2 Oral and Dental Disease Research Center, Dept. of Oral and Maxillofacial Pathology, School of Dentistry, Shiraz University of Medical Sciences, Shiraz, Iran; 3 Postgraduate, Dept. of Oral and Maxillofacial Pathology, School of Dentistry, Shiraz University of Medical Sciences, Shiraz, Iran; 4 Dept. of Oral and Maxillofacial Pathology, School of Dentistry, Shiraz University of Medical Sciences, Shiraz, Iran; 5 Dept. of Otorhinolaryngology, Khalili Hospital, Shiraz Institute for Cancer Research, Shiraz University of Medical Sciences, Shiraz, Iran; 6 Shiraz Institute for Cancer Research, Shiraz University of Medical Sciences, Shiraz, Iran

**Keywords:** Adenoid cystic carcinoma, Salivary gland tumor, Mucoepidermoid carcinoma, HSP_27_, Pleomorphic adenoma

## Abstract

**Statement of the Problem:**

Heat shock protein 27 (HSP_27_) plays important roles in many cellular processes and has been implicated in different types of diseases such as cancers.

**Purpose:**

This study aimed to evaluate the serum level of HSP_27_ in patients with salivary gland tumors and to determine its possible correlation with the prognosis of the disease.

**Materials and Method:**

This cross-sectional study was performed on 60 patients with sali-vary gland tumor including 16 pleomorphic adenoma, 33 adenoid cystic carcinoma, 6 mu-coepidermoid carcinoma, 5 acinic cell carcinoma, and 28 healthy control subjects. The con-trol cases were healthy blood donors who matched the study group in age and sex. Serum samples were obtained from the clotted blood and HSP_27_concentrations were measured with sandwich enzyme-linked immunosorbent assay (ELISA). Statistical analysis was performed by using one-way ANOVA, post Hoc test, independent sample t-test, and ROC analysis. A p value of less than 0.05 was considered as significant.

**Results:**

The mean serum level of HSP_27_ was 3956.1±3830.1 (pg/ml) in patients with malig-nant salivary gland tumor, which was significantly higher than that in benign salivary gland tumor (752.2±485.6) and healthy controls (602.3±575.8) (*p* <0.001). However, there was no significant difference in the HSP_27_ serum levels between the patients with benign salivary gland tumors and healthy controls (*p*= 0.2). No association was detected between the mean serum levels of HSP_27_ and clinicopathologic factors such as age, sex, stage and nodal metas-tasis (*p* > 0.05), except for the tumor size (*p*= 0.04).

**Conclusion:**

The HSP_27_ concentration increased in patients with malignant salivary gland tumors. Moreover, the HSP_27_ level was correlated with tumor growth, invasiveness, and diagnosability. Yet, larger clinical studies are required to explore its prognostic value.

## Introduction

Salivary gland tumors comprise 1-4% of human neoplasms [ 1 ]. Pleomorphic adenoma (PA), the most common benign salivary gland tumor, has an incidence of 70% [ 2 ]. Mucoepidermoid carcinoma (MEC) and adenoid cystic carcinoma (ACC) [ 3 ] are the most common salivary gland cancers that can metastasize to the regional lymphatics or distant organs. It has been shown that salivary gland tumors have numerous complexities in the site of occurrence, structural origins, histopathologic features, clinical appearance and prognosis [ 4 - 5 ]. Therefore, their mechanism of development and prognosis is significantly different [ 6 ]. Moreover, the salivary gland tumors have recurrence potential after treatment; thus, finding methods of early diagnosis and prevention of these tumors is very important in clinical cancers [ 6 ]. Heat shock proteins (HSPs) are a kind of important molecular chaperones expressed in the nuclei, cytoplasm, and cell membrane of both eukaryotic and prokaryotic organisms, which induce cell growth regulation and proliferation [ 7 - 8 ]. HSPs family is subdivided into four major families according to their activity including HSP_90_, HSP_70_, HSP_60_, and small molecular weight HSPs such as HSP_27_ with 27 KDa weight [ 9 ]. HSP_27_ plays important roles in many cellular processes such as cell differentiation [ 8 ], inhibition of apoptosis [ 10 ], thermotolerance, cytoskeleton dynamics, and signal transduction [ 11 ]. HSP_27_ has also been implicated in different types of diseases including cancers [ 12 ], renal injury and fibrosis [ 13 ], as well as cardiovascular and neurodegenerative diseases [ 14 ]. Over-expression of HSP_27_ has been found in different tumor types and is involved in tumor formation and development, prognosis, treatment and biological behaviors [ 15 - 16 ]. Therefore, it can be a therapeutic target and potential biomarker for diagnosis and prognosis [ 12 ]. In a recent study by VahenKepenekian *et al.* [ 17 ], it was demonstrated that HSPs overexpression in the cytosol of malignant transformed cells resulted in their translocation into the extracellular serum. The overexpression of HSP_27_ in the serum might also be the result of its release following the stress-induced death of the necrotic cells [ 17 ].

Guilan *et al.* [ 6 ] showed the overexpression of HSP_27_ in the cytoplasm of the tumoral epithelium in the salivary gland tissue and found a negative correlation between HSP_27_ tissue level and the tumor size, invasion, and metastasis. Accordingly, this study was conducted to evaluate the serum level of HSP_27_ in patients with salivary gland tumors and to determine if it has a correlation with prognosis or not. It seems that HSP_27_ can be a novel target for increasing the effect of cancer treatment; therefore, this study was designed to assess and compare the serum level of HSP_27_ in the most common benign and malignant salivary gland tumors and to consider whether its serum level was correlated with any tumor characteristics such as tumor grade and stage.

## Materials and Method

A total of 60 patients with salivary gland tumor (16 PA, 33 ACC, 6 MEC and 5 acinic cell carcinoma) and 28 healthy control subjects were engaged in this study. All the study patients were associated by ENT Department of the School of Medicine of Shiraz University of Medical Sciences. All the samples diagnosis were salivary gland tumor. The control cases were healthy blood donors who were matched with the study group based on their age and sex. The Ethics committee of Shiraz University of Medical Sciences approved this study and all the participants were informed about the nature of the study and agreed to participate by signing an informed consent form. Serum samples were obtained from clotted blood following centrifugation at 4C and stored at 80C until the time of analysis. HSP_27_ concentrations were measured with sandwich enzyme-linked immunosorbent assay (ELISA) following the manufacturer’s instructions (BMS; GmbH, Germany). 

Statistical analysis was performed by using one-way ANOVA, post hoc test, independent sample t-test, and ROC analysis. A *p* value of less than 0.05 was considered as significant. 

## Results

Table 1 shows the clinical characteristics of 44 patients with malignant salivary glands. The mean age of patients with malignant salivary gland tumor was 51.4±17.2 years old (ranging 21-80). The mean age of patients with benign tumors was 45.6±14.6 years old (ranging 22-71) and healthy controls were 49.7±17.2 years old (ranging 19-85). The mean serum level of HSP_27_ was 3956.1±3830.1 (pg/ml) in patients with malignant salivary gland tumor, which was significantly higher than that in those with benign salivary gland tumor (752.2± 485.6) and healthy controls (602.3±575.8) (*p* <0.001). The serum levels of HSP-27 in patients with benign salivary gland tumors and control groups had not significant differences (*p*= 0.2) (Table 2). The ROC curve analysis showed that the optimal cut-off value for HSP_27_ was 809.01(pg/ml), indicating that HSP_27_ serves as a diagnostic marker for malignant salivary gland tumors. This yielded a sensitivity of 75% and specificity of 75%. There was no correlation between the mean serum levels of HSP_27_ and clinicopathologic factors such as age, sex, stage and nodal metastasis (*p* > 0.05), except for the tumor size (*p*= 0.04). The serum level of HSP_27_ was significantly higher in patients with larger tumor size than the small tumor size. 

**Table 1:** The base line data of all groups

Patients	Number	Age( Mean (range))
Benign tumors	16	45.6±14.6 (22-71)
Malignant tumors	44	51.4±17.2 (21-80)
Control	28	49.7±17.2(19-85)
Total	60	48.3±16.2 (20-78)

**Table 2:** Clinicopathologic features and the mean HSP_27_ level of the patients with malignant salivary gland tumor

Clinicopathologic data	Number (%)	Mean HSP_27_level±SD (pg/ml)	*p* Value
Tumor Size			0.04
T1+T2	32(26.7)	3011.6±3426.5	
T3+T4	12(26.7)	6477.1±6268.5	
Lymph node involvement			0.5
N0	28(63.6)	3701.1±4111.7	
N1	16(36.4)	4402.4±3359.9	
Distant Metastasis			0.08
M0	41(93.1)	3742.6±3549.9	
M1	3(6.9)	6874.1±7046.9	
Stage			0.3
I+ II	20(45.5)	3117.7±3495.05	
III+ IV	24(54.5)	4654.8±4026.8	

## Discussion

Biomarkers are used to detect and monitor the growth of the tumor and evaluate the effect of anti-cancer therapies [ 18 ]. A major disadvantage of tissue-based biomarkers is their limited accessibility and the risk of developing infection due to invasive surgery. Therefore, blood-derived biomarkers are better than biopsies in detecting and monitoring the tumor in that these samples can be taken before, during and after treatment with minimal invasion [ 19 ]. 

HSPs are a series of highly conserved proteins that play a crucial role in maintaining protein homeostasis [ 20 ]. HSPs are produced under stressful conditions and they participate in folding and assembling the proteins [ 12 ]. However, HSPs can be secreted in the blood circulation and have been shown to interact with some immune cells [ 21 ]. It has been demonstrated that HSP_27_ expression is crucial for tumor development and is involved in tumor behavior and prognosis [ 22 - 23 ]. Increased HSP_27_ expression has been reported in different cancers [ 24 - 25 ] and is associated with poor prognosis in the liver, prostate, and gastric carcinoma [ 26 - 27 ]. 

In the present study, circulation of HSP_27_ level was obviously upregulated in patients with malignant Salivary gland tumors than in those with benign tumors and healthy control subjects. This was in line with those of the previous studies that evaluated the HSP_27_ level in breast cancer, non-small cell lung cancer, pancreatic and

gastric adenocarcinoma [ 28 - 31 ]. This study demonstrated that HSP_27_ level can be use-d as a discriminating biomarker for differentiating patients with and without cancer. This was consistent with the results of Lia *et al.* [ 27 ] and De *et al.* [ 32 - 33 ]. Lia *et al.* [ 27 ] stated that Heat shock protein 27 levels were significantly higher in cancer and pancreatitis compared with control (*p* < 0.001 for both) and De *et al.* [ 32 - 33 ] reported that the use of HSP 27 ELISA could be extremely useful in evaluating the role of soluble HSP 27 in breast or other cancers.

Cell damage or necrotic cell death can cause the release of intracellular HSP into circulation that could lead to elevated HSP serum concentration [ 34 ]. In this study, HSP elevation in patients with malignant salivary gland tumor might be related to its increased production and release because of cell damage. In salivary gland carcinoma, HSP_27_ level did not correlate with prognostic factors, except for the tumor size. In a recent study on non-small cell lung cancer, it was found that the serum level of HSP_27_ was significantly elevated in patients at the advanced stage than the early stages [ 35 ]. However, no significant relationship was found between the serum level of HSP_27_ and histological types and sex [ 35 ].

Van Eden *et al.* [ 36 ] showed that HSP released by the cells were biologically active molecule, confirming the hypothesis that elevated circulating HSP_27_ level in the serum can lead to tumor growth and invasiveness. Different mechanisms have been proposed to explore the role of HSP_27_ in tumor progression. Furthermore, it has been shown that HSP_27_ has the ability to block apoptotic cell death and may contribute to tumor growth via inhibition of apoptosis [ 37 ]. It can also directly interact with other pro-survival proteins and regulate the cell survival [ 38 ]. 

In a recent study by Banerjee *et al.* [ 30 ], it was reported that increased HSP_27_ levels in human breast tumor could induce unresponsiveness in T cells. Moreover, H-SP induced significant neovascularization via macrophage differentiation, and was critically important for tum-or growth [ 30 ]. They suggested that immune suppression induced by HSP_27_ was a new tumor growth supporting mechanism that might lead to novel treatment strategies by targeting the released HSP_27_ [ 30 ]. Furthermore, our results showed the positive association of HSP_27_ serum levels with tumor size; this finding suggests that the serum level of HSP_27_ is related to disease condition in salivary gland tumors. It might be suggested that the mechanism of HSP_27_ through angiogenesis may lead to the growth of the tumor. Hsu *et al.* [ 11 ] demonstrated that HSP_27_ overexpression was associated with resistance to chemotherapy. Resistance to chemotherapy and ra-diotherapy has been reported in malignant salivary gland tumor [ 39 ]. Resistance to chemotherapy, in salivary gland cancer may partly contribute to elevated HSP_27_. 

This study could not evaluate the expression of HSP_27_ and the relationship between HSP_27_ expression and its serum level. Therefore, further studies are required to explore this relationship. 

## Conclusion

This study showed that HSP_27_ concentration increased in patients with malignant salivary gland tumors and besides its diagnostic ability, its level was correlated with tumor growth and invasiveness. Nevertheless, larger clinical studies are required to verify its prognostic value. 

## Acknowledgement

The authors thank the Vice-Chancellery of Shiraz University of Medical Sciences for supporting this research (Grant #92-01-03-6495). This manuscript was based on the thesis by Negar Moghadasi for partial fulfillment of DDS degree. The authors thank Dr. M. Vossoughi of the Dental Research Development Center of the Dental School, for the statistical analysis.

## Conflict of Interests

 The authors declare that they have no conflict of interest.
